# Minimally invasive osteosynthesis of adult tibia fractures by means of rigid fixation with anatomic locked plates

**DOI:** 10.1007/s11751-013-0164-9

**Published:** 2013-07-24

**Authors:** Melih Güven, Emrah Ceviz, Murat Demirel, Turhan Özler, Onur Kocadal, Ayberk Önal

**Affiliations:** 1 FEBOT, Department of Orthopaedics and Traumatology, Faculty of Medicine, Yeditepe University, Istanbul, Turkey; 2 Department of Orthopaedics and Traumatology, Çankırı Government Hospital, Çankırı, Turkey; 3 Department of Orthopaedics and Traumatology, Ankara Bayındır Hospital, Ankara, Turkey

**Keywords:** Tibia fracture, Minimally invasive, Osteosynthesis, Biological, Locked plate, Screw

## Abstract

Main principle of biological fixation by minimally invasive locked plate osteosynthesis (MILPO) in lower extremity long bone fractures is relative stability which is provided by using long plate with limited number of screws. Some biomechanical studies have been reported about this issue. However, clinical studies are still missing. The aims of this retrospective extended case series were to evaluate the clinical and radiological results of adult tibia fractures treated by MILPO and the effect of plate length and screw density on complication rates. Twenty tibia fractures in 19 patients (mean age 42.3 years) operated by MILPO were reviewed. According to the AO classification, diaphyseal and metaphyseal fractures without intraarticular extensions were simple and wedge-type fractures, whereas all intraarticular fractures were comminuted. Number of screws, cortices and empty screw holes proximal and distal to the fracture, plate-span ratio (plate length divided by overall fracture length), plate-screw density (number of inserted screws divided by number of plate holes), fixation failures, delayed or nonunion, malalignment and leg length discrepancy were documented. Mean follow-up was 16 (range 12–26) months. On average, 4 screws with 6 cortices were used both proximally and distally in all fractures. Only in diaphyseal fractures, one screw hole close to the fracture was omitted. Average plate-screw density and plate-span ratio were 0.68 and 4, respectively. Mean union time was 3 months. There were no cases of delayed or nonunion on the final follow-up. Plate bending was observed in one patient who had fair result. The remaining 18 (94.8 %) patients showed good and excellent results. Satisfactory results can be achieved despite low plate-span ratio and high plate-screw density in simple and wedge-type diaphyseal fractures of the tibia. Additionally, plate-screw density can be higher at metaphysis in intraarticular fractures, in which essential point is a perfectly stable fixation that provides early motion.

## Introduction

Biological fixation by minimally invasive locked plate osteosynthesis (MILPO) has become an option for treating of lower extremity fractures. It has well-documented biological advantages compared to conventional plate osteosynthesis including reduced tissue devitalization, avoidance of iatrogenic damage of blood supply around the fracture and early fracture union with decreased wound complications [1, 2]. Several previous studies [3–9] have reported high success rates with this technique in complex periarticular fractures as well as comminuted metaphyseal and diaphyseal fractures.

The basic principles of this technique include indirect closed reduction, extraperiosteal dissection, anatomic alignment and relative stability which permits limited motion at the fracture site and creates secondary bone healing with callus formation [10]. The amount of this elastic motion is determined by the length, cross-sectional area and material properties of the plate and the density and diameter of the inserted screws as well as the use of unicortical or bicortical screws [2, 11]. Plate length and screw density are key factors for the stability of fixation [10]. Some in vitro biomechanical studies [12, 13] have reported recommendations that long plates with limited number of screws are essential to obtain a sound, flexible fixation and to reduce implant failure. However, clinical studies supporting this practice are lacking.

We retrospectively reviewed patients with tibia fractures treated by MILPO and evaluated the outcomes regarding clinical and radiological results as well as the effect of plate length and screw density on complication rates.

## Materials and methods

The patient database in the first author’s institution was examined in the period March 2008–June 2010 for patients who had undergone surgery for lower extremity fractures. Hospital records and radiographs were reviewed, and only the patients operated by MILPO for tibia fractures were included in the study. Pathological fractures secondary to tumour, metabolic bone disease and malleolar fractures were excluded.

Nineteen patients (20 tibia fractures) who had a minimum follow-up period of 1 year were identified. None of the patients were lost to follow-up. All patients were informed about the study which was approved by the institution’s human subject review board.

Anteroposterior and lateral radiographs had been obtained to establish the fracture pattern, classification and surgical planning. Twelve (63 %) of 19 patients had other injuries (Table 1). Five of the 20 tibial fractures were located proximally, ten diaphyseal and five distally. All fractures were classified according to the AO classification system [14] (Table 2). None of the patients had neurovascular deficit.

**Table 1:** Additional trauma in patients and treatment modalities

Type of additional trauma	*n*	Treatment
Ipsilateral nondisplaced patella and femur medial condyle fractures	1	Percutaneous screw fixation
Humerus fracture at the distal diaphysis	1	Fixation with uniplanar external fixator
Fibula fracture at the same level	6	Conservative treatment in two patients, open reduction and internal fixation in four patients
Fibula fracture at the different level	3	Conservative treatment
Ipsilateral posterior hip dislocation	1	Closed reduction

*n* number of patients

**Table 2:** AO classifications of the fractures and types of plates used in the study

Fracture localization	AO classification	*n*	Type of plate used
Proximal	41-B1	1	3.5 mm proximal tibia anatomic medial LP
	41-C1	3	3.5 mm proximal tibia anatomic medial LP
	41-C2	1	3.5 mm proximal tibia anatomic medial LP
Diaphyseal	42-A1	3	3.5 mm distal tibia anatomic medial LP
	42-A2	2	3.5 mm distal tibia anatomic medial LP
	42-B1	3	3.5 mm distal tibia anatomic medial LP
	42-B2	2	3.5 mm proximal tibia anatomic medial LP
Distal	43-A1	2	3.5 mm distal tibia anatomic medial LP
	43-C1	3	3.5 mm distal tibia anatomic medial LP

*n* number of applications, *LP* locking plate

### Operative method

All patients were treated by the same surgical team in the first author’s institution. The average time between the injury and surgical procedure was 11.3 h (range 2–24). Patients with open fractures were operated within an average of 8.8 h following injury (range 2–12). All operations were performed on a radiolucent table in a supine position without using a traction table. Debridement combined with irrigation was repeated in the operating room for open fractures. The main fracture fragments were aligned using manual traction and closed reduction manoeuvres. Reduction in large fragments in one comminuted fracture (AO 43-C1) and in one spiral-oblique simple fracture (AO 42-A1) was maintained by percutaneously inserted individual lag screws. In three patients (AO 43-C1), the articular fragments required open reduction and rigid fixation before plate placement. Nine patients with tibial fractures had fractures on the fibula also. Four fibular fractures at the level of the syndesmosis were treated by open reduction and internal fixation using anatomic fibular plates to provide lateral column stability and restoration of the correct length.

**Fig. 1:**
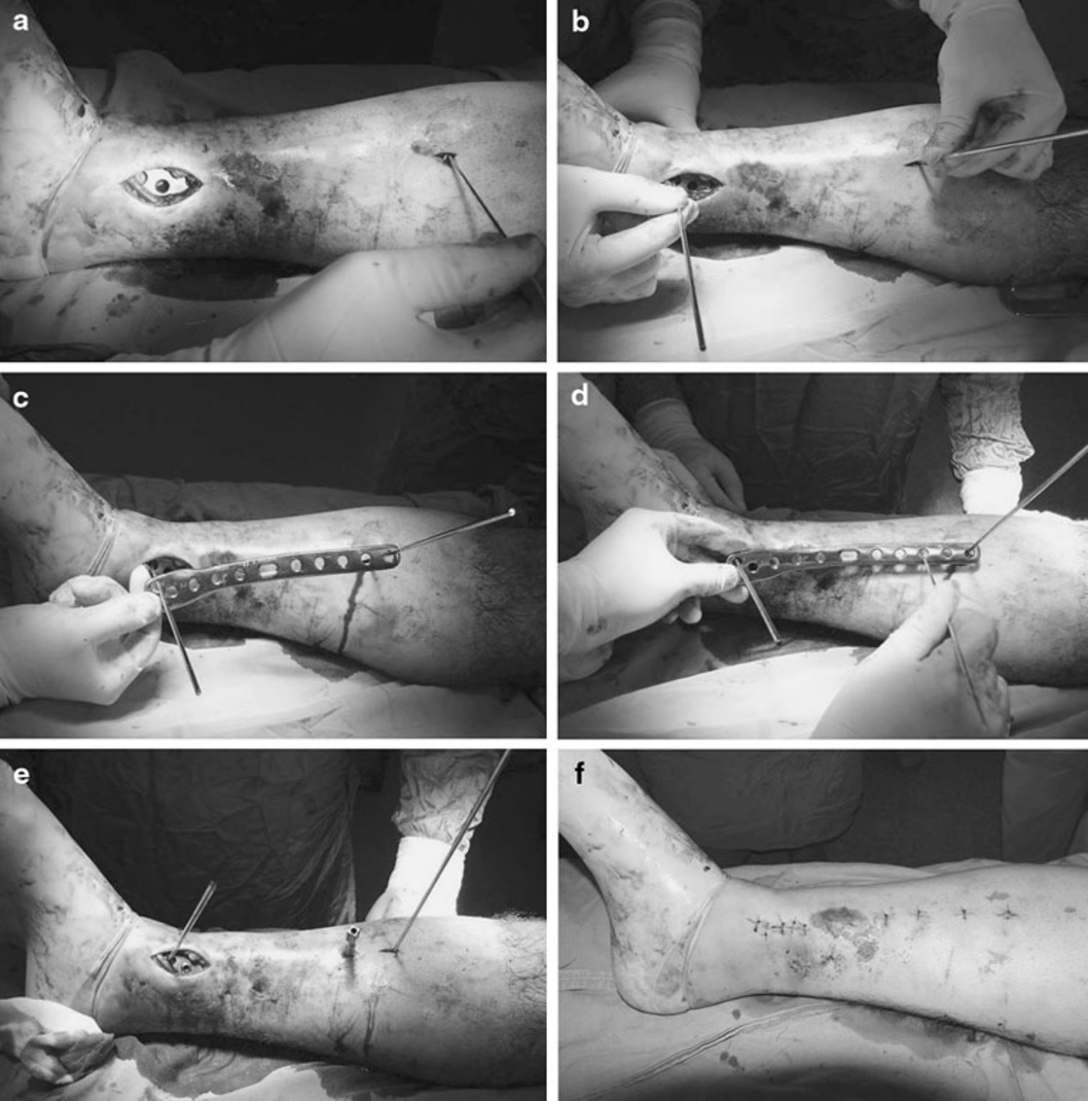
Screw fixation technique used in the study. Kirschner wires are inserted into the bone through the most proximal and distal holes of the plate (**a** and **b** ). A second plate of similar size and length is placed through the same holes on the Kirschner wires and used as an external guide to localize the screw holes (**c** and **d**). The locking drill guide is inserted into the screw hole (**e**). Appearance of the leg after skin closure (**f**)

The plate was applied on the anteromedial aspect of the tibia for all fractures. A 3–4-cm skin incision was made proximal or distal to the fracture. An extraperiosteal, subcutaneous tunnel was created with a periosteal elevator. Pre-contoured 3.5 mm proximal or distal anatomic medial locked plates (TST; Turkish Spinal Trauma Medical Devices; Istanbul; Turkey), which included both locking and compression screw holes, were used and passed along this tunnel (Table 2). Once satisfactory plate positioning was achieved, the plate was secured by passing 3-mm Kirschner wires through the most proximal and distal holes. A second plate of similar size and length was placed using the same holes on the Kirschner wires. This acted as an external guide to localize the screw holes and skin incisions without need of fluoroscopic control [16] (Fig. 1). One proximal and distal screw was inserted. Additional screws were then applied using the same technique. In general, locking screws were used in the juxta-articular and diaphyseal segments, while nonlocking screws were selected for reduction in large fragments as lag screws. None of the screws in the plate were applied in compression mode. Primary bone grafting was not performed in any cases.

The number of screws used proximal and distal to the fracture as well as the number of proximal and distal cortices engaged by screws was documented. Empty screw holes proximal and distal to the fracture zone were also documented. The plate-span ratio, which is described as the quotient of plate length divided by overall fracture length, and the plate-screw density, which is described as the quotient of inserted screws divided by number of plate holes, were recorded [2, 10]. Fracture length was determined as the vertical distance between the most proximal and distal points of the fracture line on the radiographs.

Patients were not splinted post-operatively. On the second post-operative day, active and passive knee and ankle motion were encouraged and straight leg lifts were started. With the exception of the patient with bilateral tibia fractures, all patients were allowed to walk with crutches but without weight-bearing. Progressive weight-bearing was encouraged at the sixth week, and full weight-bearing was allowed for patients with the ability to walk without pain and/or for patients with callus formation on radiographs.

Routine clinical and radiological assessments were performed at 3 and 6 weeks, also at 3, 6, 9 and 12 months. Bone union was defined clinically as the absence of pain and instability at the fracture site and radiographically as the presence of bridging across two cortices on both anteroposterior and lateral views. A fracture in the process of union but not united at 6 months was considered as a delayed union. A nonunion was defined as the absence of progressive fracture healing for three consecutive months extending beyond 6 months from injury [6]. Complications of fixation failure such as plate bending, plate fracture, locking screw failure and skin irritation, infection and secondary surgical procedures were also documented.

Angular and rotational deformities as well as leg length discrepancy were recorded at the final follow-up. Satisfactory alignment was defined as <5° of angular deformity in either the coronal or sagittal planes [17]. In addition, shortening of >1 cm and rotational mismatch >10° (assessed clinically) were documented. On the final follow-up, the outcome results were evaluated according to Johner–Wruhs’ criteria [18] which take functional, clinical, radiological and subjective outcomes into account. The patients were graded into excellent, good, fair and poor on this scale depending on pain, gait, range of motion at the knee, ankle and subtalar joints, deformity, and ability to do daily work.

## Results

There were five (26 %) females and 14 (74 %) males. The average age of the patients at the time of surgery was 4.3 years (range 18–86). The right lower limb was injured in nine patients, left in nine patients and both limbs were injured in one patient. There were three (15.8 %) cases (AO 42-A1, 42-B1 and 43-C1) of grade II and two (10.5 %) cases (AO 41-B1 and 41-C2) of grade IIIA open fractures according to Gustilo and Anderson classification [15]. Immediate debridement and irrigation were performed in the emergency room and appropriate antibiotic therapy started for these fractures.

The patients were followed up for a mean of 16 months (range 12–26 months). On average, 4 screws (range 3–6) were used both proximally and distally to the fracture site. Six cortices (range 3–12) were fixed both proximally and distally on average. In proximal and distal metaphyseal (intraarticular as well as extra-articular) fractures, there were no empty screw holes close to the fracture zone. However, in all diaphyseal fractures, one screw hole close to the fracture zone was omitted. The average plate-screw density and plate-span ratio were 0.68 (range 0.5–1) and 4 (range 1.43–9.4), respectively (Table 3).

**Table 3:** The number of screws and cortices on either side of the fracture, plate-screw density and plate-span ratio in the study according to the fracture localization

Fracture localization (Types of fractures)	*n*	nPS*	nDS*	nPC*	nDC*	PSD*	PSR*	Number of complications
Proximal metaphyseal intraarticular (Type B and C)	5	4	3.7 (3–5)	4.7 (4–6)	6.2 (4–8)	0.76 (0.57–1)	2.38 (1.8–3.5)	–
Diaphyseal (Type A and B)	10	4	4.1 (3–6)	7 (4–8)	6.8 (3–12)	0.66 (0.5–0.83)	5.15 (3.5–9.4)	1
Distal metaphyseal extraarticular (Type A)	2	3.5 (3–4)	4.5 (4–5)	6.5 (5–8)	6	0.67 (0.64–0.7)	2.5	–
Distal metaphyseal intraarticular (Type C)	3	3.6 (3–4)	4.6 (4–5)	7.3 (6–8)	5.3 (4–7)	0.62 (0.57–0.66)	1,43	–

*n* number of applications, *nPS* number of proximal screws, *nDS* number of distal screws, *nPC* number of proximal cortices, *nDC* number of distal cortices, *PSD* plate-screw density, *PSR* plate-span ratio

* Values are given as the mean with the range in parenthesis

**Fig. 2:**
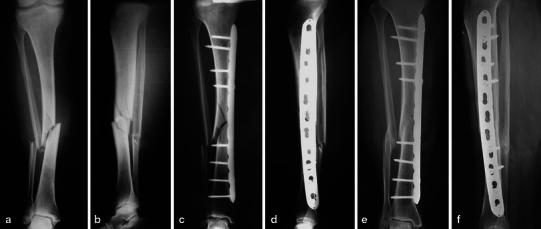
Preoperative anteroposterior (**a**) and lateral (**b**) radiographs of 56-year-old female patient show a wedge type (AO 42-B1) right tibia fracture and associated fibula fracture at the same level. Tibia fracture was fixed with a 4.5 mm locking compression plate (**c** and **d**). One proximal screw hole close to the fracture was omitted. Plate-span ratio was 4.6 and plate-screw density was 0.66. 18 months postoperatively, anteroposterior (**e**) and lateral (**f**) radiographs showed uneventfully healing of the fracture without any complications

All patients were allowed partial weight-bearing at the end of the sixth week. The average time to full weight-bearing was 7.7 weeks (range 6–12). The average time to union was 3 months (range 3–4) (Fig. 2). Open tibia fractures in five patients were healed radiographically on the third month.

There were no wound complications; this included superficial or deep infections and vascular or neurological problems in the early and late post-operative periods. There were no cases of delayed or nonunion. Plate bending was observed in one patient (AO 42-A1) due to uncontrolled weight-bearing in the third post-operative week. The plate-screw density and plate-span ratio of this patient were 0.7 and 6, respectively. A fair result was observed in this patient with 15° valgus deformity and 1-cm extremity shortening on the final follow-up. None of the patients showed rotational deformity. All patients had full range of motion of their knee and ankle joints compared to the opposite uninjured side.

On the final follow-up one (5.2 %) patient had a fair result, four (21.1 %) patients had good results and 14 (73.7 %) patients had excellent results according to Johner–Wruhs’ criteria. No patients with poor results were recorded.

## Discussion

Biological fixation with percutaneous bridge plating technique has three important components including closed-indirect reduction, minimal soft tissue dissection and stabilization with a long percutaneously inserted plate fixed with a limited number of widespread screws [10]. If the length of the plate increases, the pull-out force acting at the screw decreases [2]. It has been recommended that the length of the locked plate should be 8–10 times the length of the fracture in simple patterns and 2–3 times the length in comminuted fracture patterns [2, 10]. Although two unicortical screws on each main fragment are the minimal requirement to keep the construct stable, Hertel et al. [19] advised to fixate at least three cortices on either side of the fracture secondary to their clinical observation of radiolucency at the screw–bone interface. Current recommendations are at least two screws with three cortices per main fragment for simple fractures and at least two screws with four cortices per main fragment for comminuted fractures [10]. Additionally, a screw bone density <0.5 is recommended to decrease the bending moments experienced at the most proximal and distal screws. In simple fractures, one or two screw holes should be omitted on each side of the fracture to allow motion and initiate spontaneous secondary fracture healing with callus formation. However, in comminuted fractures, placement of innermost screws as close as possible to the fracture is recommended [12].

In a series consisted of 169 fractures in 144 patients who were treated with locking compression plates (LCP) using a MILPO technique, Sommer et al. [20] reported 27 (16 %) complications including 5 cases of implant loosening and 4 cases of implant breakage. They concluded that these implant failures were related to intraoperative technical errors including the use of plates that were too short and those that did not have adequate empty screw holes over the fracture site.

However, in the stabilization of long bone fractures by the bridge plating technique, there is a fine line between flexible fixation, which enables callus formation and improves the healing process, and an unstable fixation, which can lead to nonunion and/or implant failure. Stability determines the amount of strain at the fracture site, and fracture gap strain determines the type of healing that can occur at the fracture site. Primary bone healing occurs when the strain is kept to <2 %, a scenario of absolute stability at the fracture site, whereas secondary bone healing characterized by callus formation occurs when the strain is kept between 2 and 10 % which comprises a relative stability. If the strain is >10 %, bone cannot be formed [11].

There are some clinical reports in the literature about the disadvantages of MILPO technique including prolonged time to fracture union and full weight-bearing. Gupta et al. [6] reported the results of 79 patients with distal metaphyseal tibia fractures operated by MILPO. They used 4.5-mm limited-contact dynamic compression plates (LC-DCP) and LCP. No incidence of nonunion or delayed union was observed in cases where plating was done in compression mode, but all the instances of delayed union (seven cases) or nonunion (three cases) occurred with bridge mode plating. Hasenboehler et al. [4] retrospectively evaluated the healing pattern and the clinical evolution of diaphyseal and distal tibial shaft fractures over two and a half years in 32 patients. They used 4.5-mm LCP and 3.5-mm LCP-pilon form plates. Plate bending was observed in one patient and called for reoperation at fifth month. Two patients required reoperation at thirteenth month secondary to pseudoarthrosis. They observed a prolonged healing time (average at 24 weeks; range 12–36 weeks) particularly in simple fracture patterns (Type A) when a bridge plating technique was used. Therefore, they concluded that if percutaneous plating using MILPO technique was planned for a simple fracture, a compression osteosynthesis with percutaneous interfragmentary lag screws and neutralization plate should be performed. If not possible, they advised formal open reduction and internal fixation.

In the present study, diaphyseal as well as metaphyseal fractures without intraarticular extensions were simple (Type A) and wedge (Type B)-type fractures according to the AO classification. Plate-screw density was over 0.5 in all of them, and plate-span ratio changed between 2.5 and 5.15. Only one implant-related complication (plate bending) was observed but without delayed or nonunion. All comminuted fractures (Type C) in the present study were intraarticular. Plate-screw density was also high in these fractures with acceptable plate-span ratio changed between 1.5 and 2.5. Although it has been concluded that the plate-screw density should be under 0.5 at the diaphyseal region and zero at the fracture zone, we conclude that high plate-screw density increases the stiffness of fracture fixation and reduces therefore the rates of delayed and nonunion in simple and wedge-type fractures. Plate-screw density is higher at the metaphyseal–epiphyseal areas as the most important objective is to obtain sufficient stability that enables early joint motion [2, 10, 11].

There is no clear consensus in the literature about the time to weight-bearing after biological fixation using bridge plating. While some authors [3, 8, 21] have recommended partial weight-bearing within the first 3 weeks post-operatively with or without using brace or splints, others [4, 5, 22] have not allowed weight-bearing for a minimum period of 6–7 weeks. In the present study, we restricted of progressive weight-bearing for 6 weeks. As plate bending was observed in one patient with tibia fracture due to uncontrolled weight-bearing in the third post-operative week, we advise restriction of weight-bearing in the early post-surgical period, particularly in simple and wedge-type diaphyseal fractures with a low plate-span ratio and high plate-screw density.

Our study is a consecutive series by a single surgical team with experience in minimally invasive plating. However, there are some limitations of our study including its retrospective nature, the lack of a control group and the small number of patients. Only one patient had complications in the present study. Therefore, we could not compare the results between the patients with and without complications by the variables of plate length and screw density. This issue requires a larger number of patient cohort.
